# Supplementary figures and images for: Plural molecular and cellular mechanisms of pore domain KCNQ2 encephalopathy
Source: eLife. 2025 Jan 6;13:RP91204. doi: 10.7554/eLife.91204 (PMC11703504; doi:10.7554/eLife.91204)

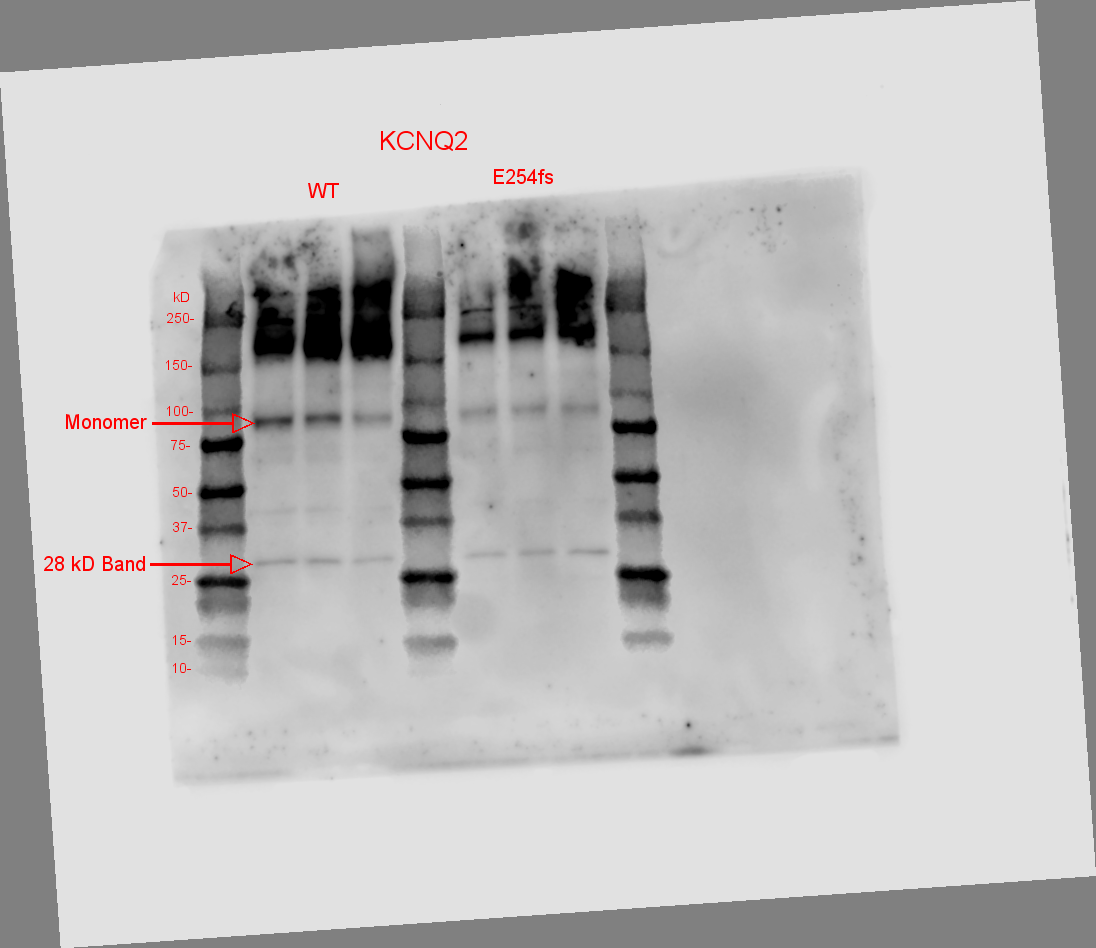

Supplement: Figure 4—figure supplement 2—source data 2. [file elife-91204-fig4-figsupp2-data2.zip › Figure 4-figure supplement 2-source data 2-Uncropped and labeled blot/Figure 4-S2-Source Data 2.tif]

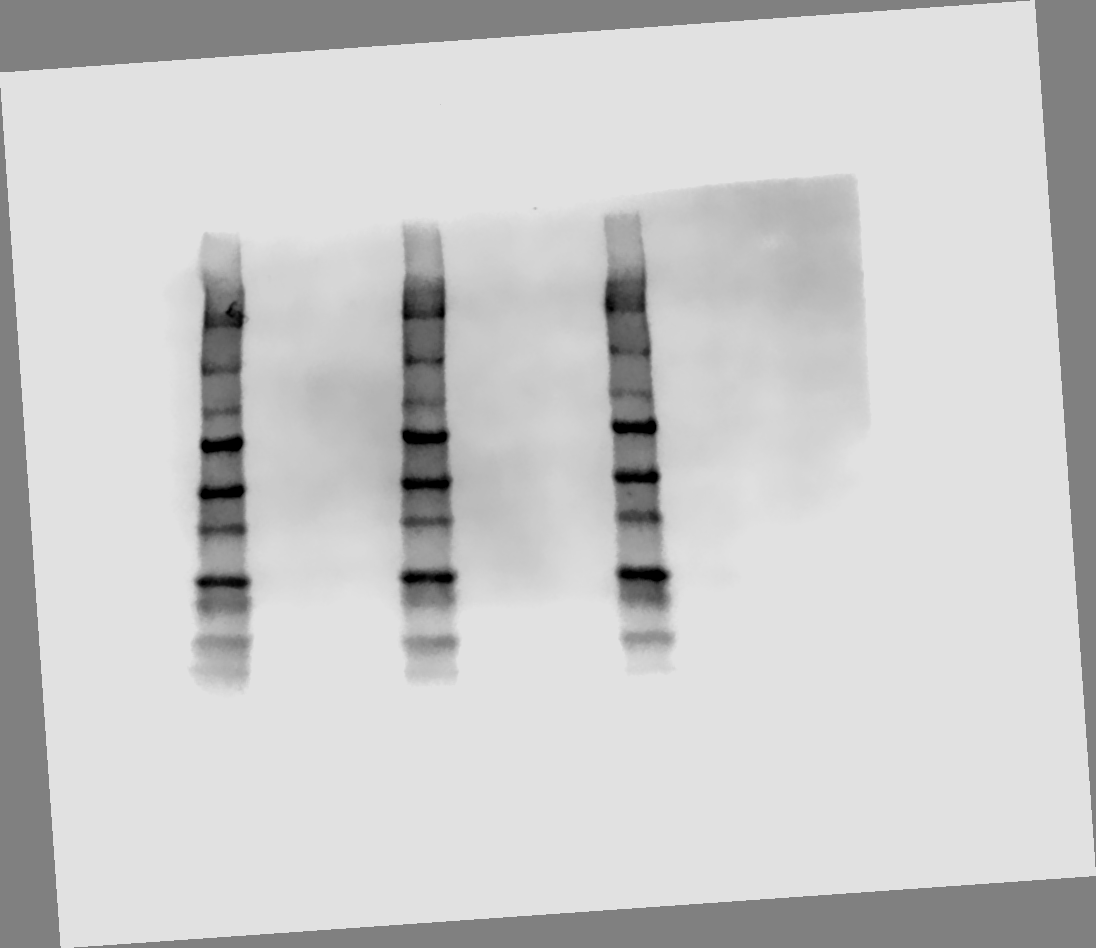

Supplement: Figure 4—figure supplement 2—source data 3. [file elife-91204-fig4-figsupp2-data3.zip › Figure 4-figure supplement 2-source data 3-Raw unedited blot/Figure 4-figure supplement 2-Source Data 3-Uncropped blot 700 nm.tif]

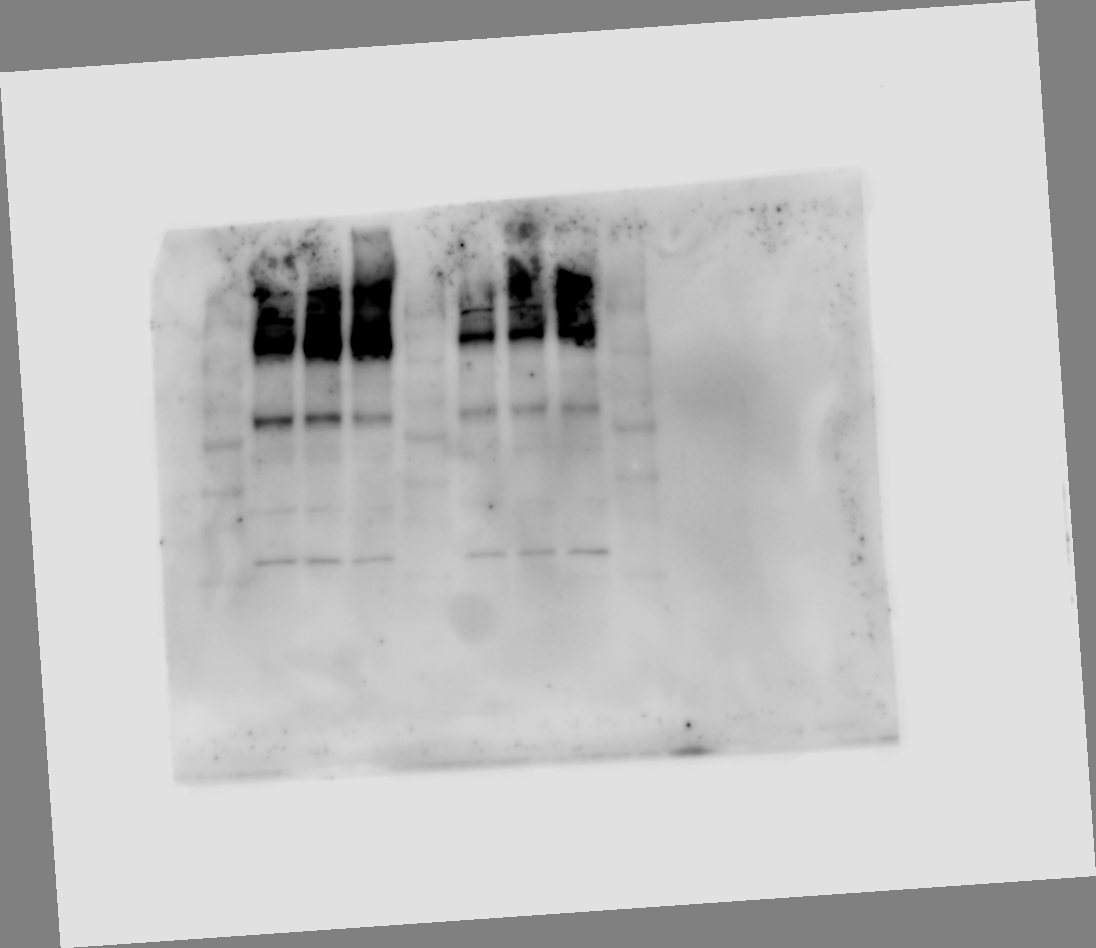

Supplement: Figure 4—figure supplement 2—source data 3. [file elife-91204-fig4-figsupp2-data3.zip › Figure 4-figure supplement 2-source data 3-Raw unedited blot/Figure 4-figure supplement 2-Source Data 3-Uncropped blot ECL.tif]

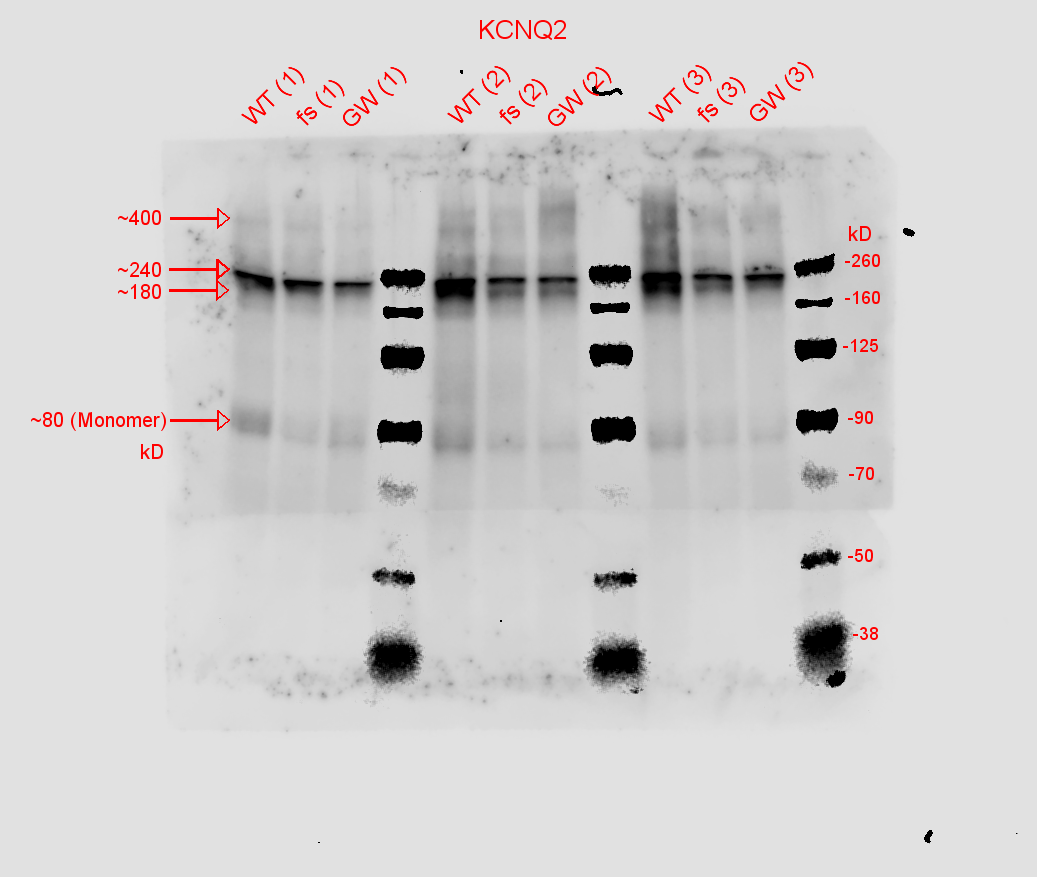

Supplement: Figure 9—source data 2. [file elife-91204-fig9-data2.zip › Figure 9-source data 2-Uncropped and labeled blots/Figure 9-Source Data 2-KCNQ2.tif]

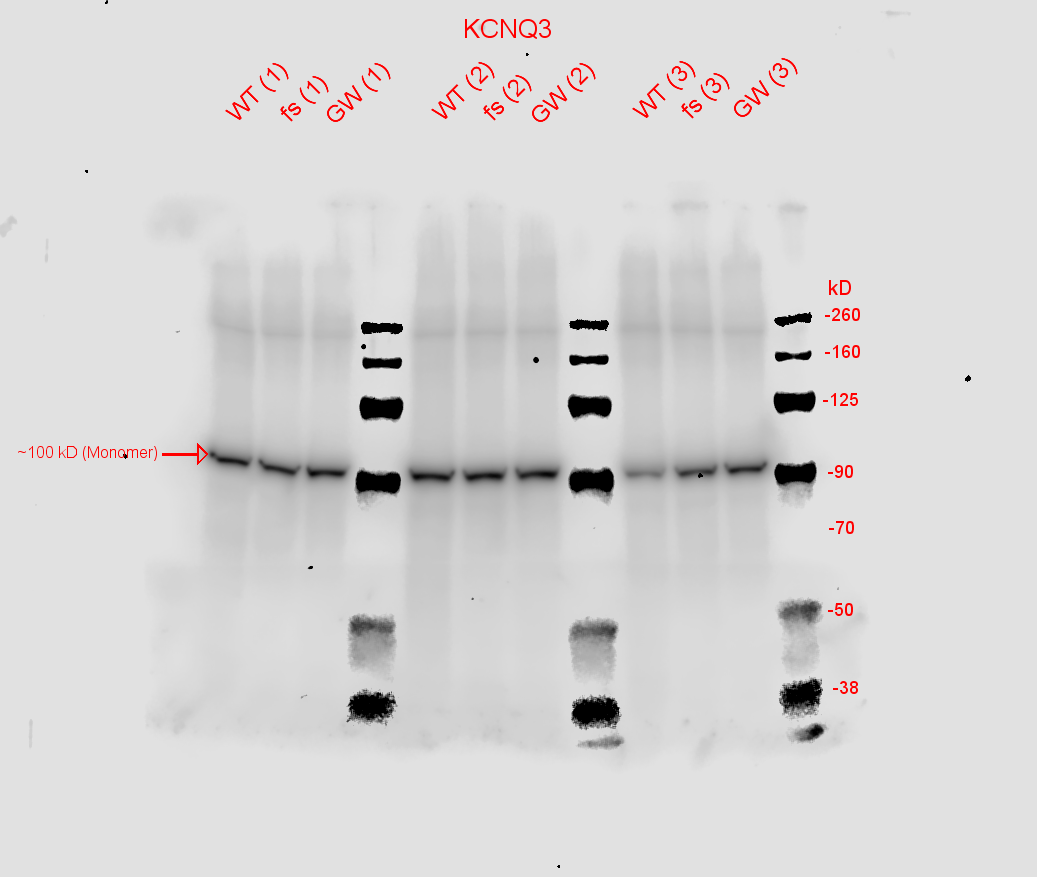

Supplement: Figure 9—source data 2. [file elife-91204-fig9-data2.zip › Figure 9-source data 2-Uncropped and labeled blots/Figure 9-Source Data 2-KCNQ3.tif]

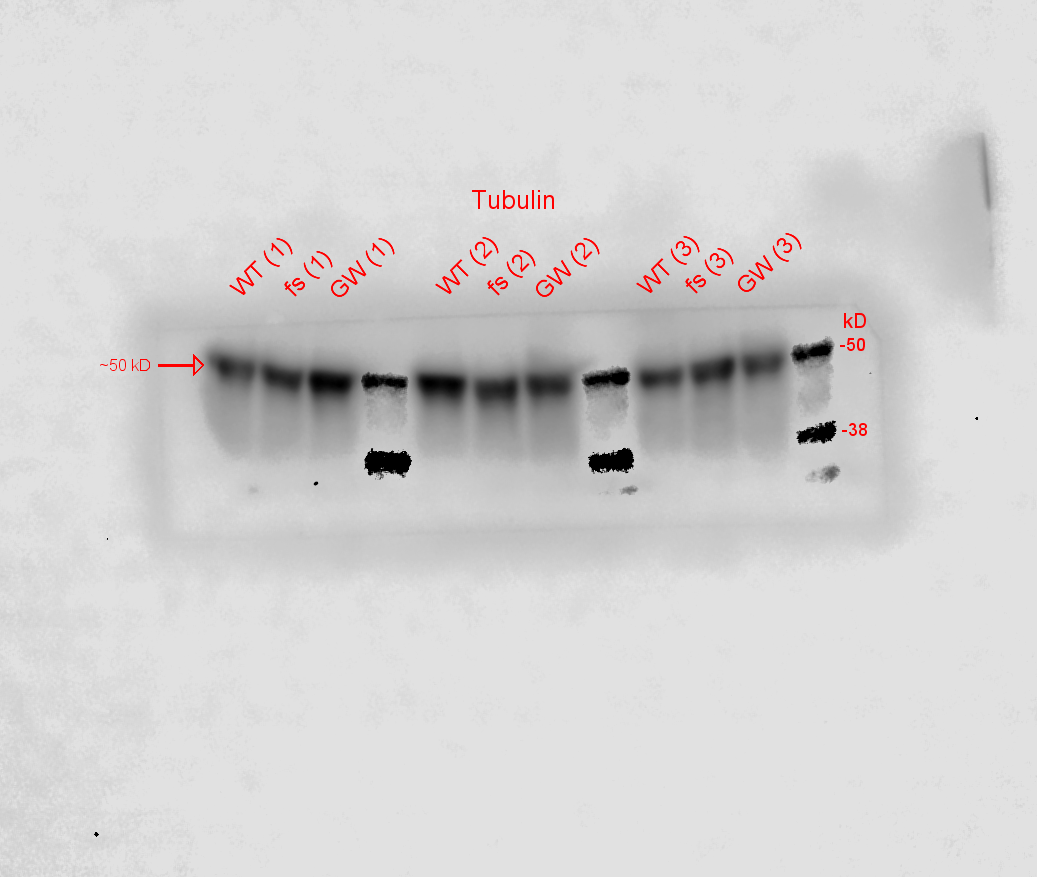

Supplement: Figure 9—source data 2. [file elife-91204-fig9-data2.zip › Figure 9-source data 2-Uncropped and labeled blots/Figure 9-Source Data 2-Tubulin.tif]

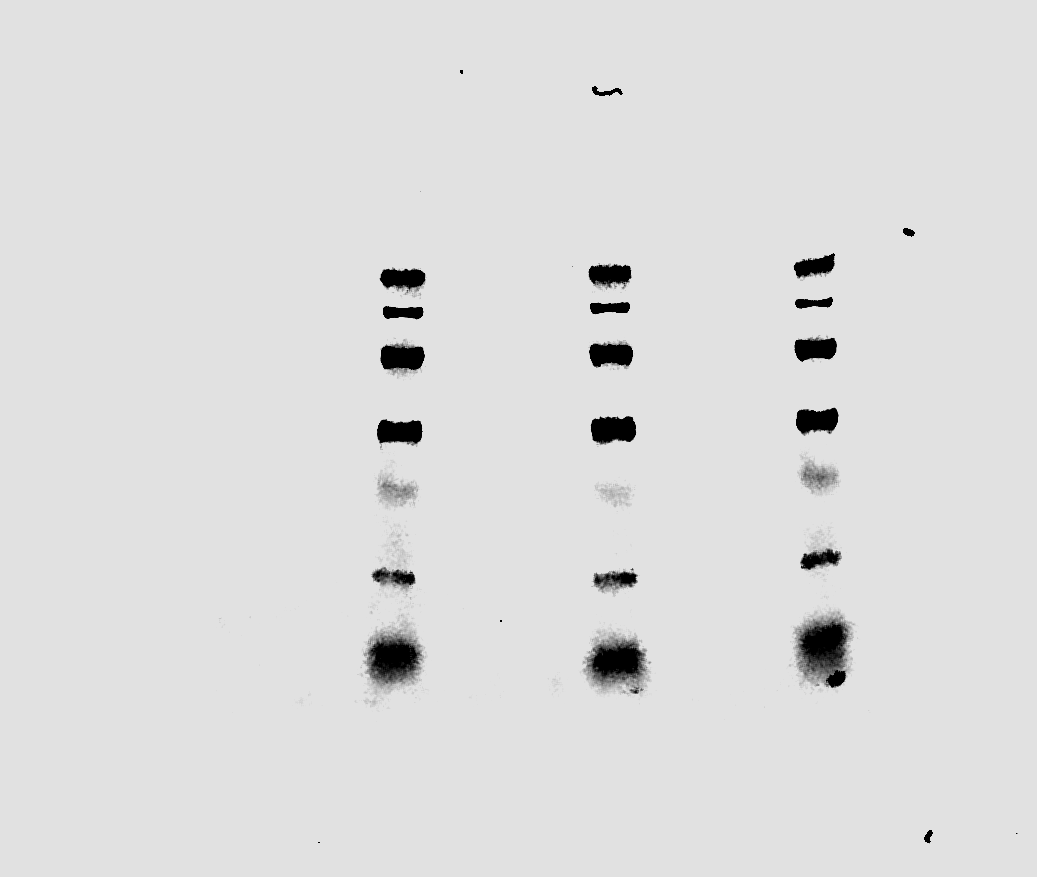

Supplement: Figure 9—source data 3. [file elife-91204-fig9-data3.zip › Figure 9-source data 3-Raw unedited blots/Figure 9-Source Data 3-KCNQ2 MW stds.tif]

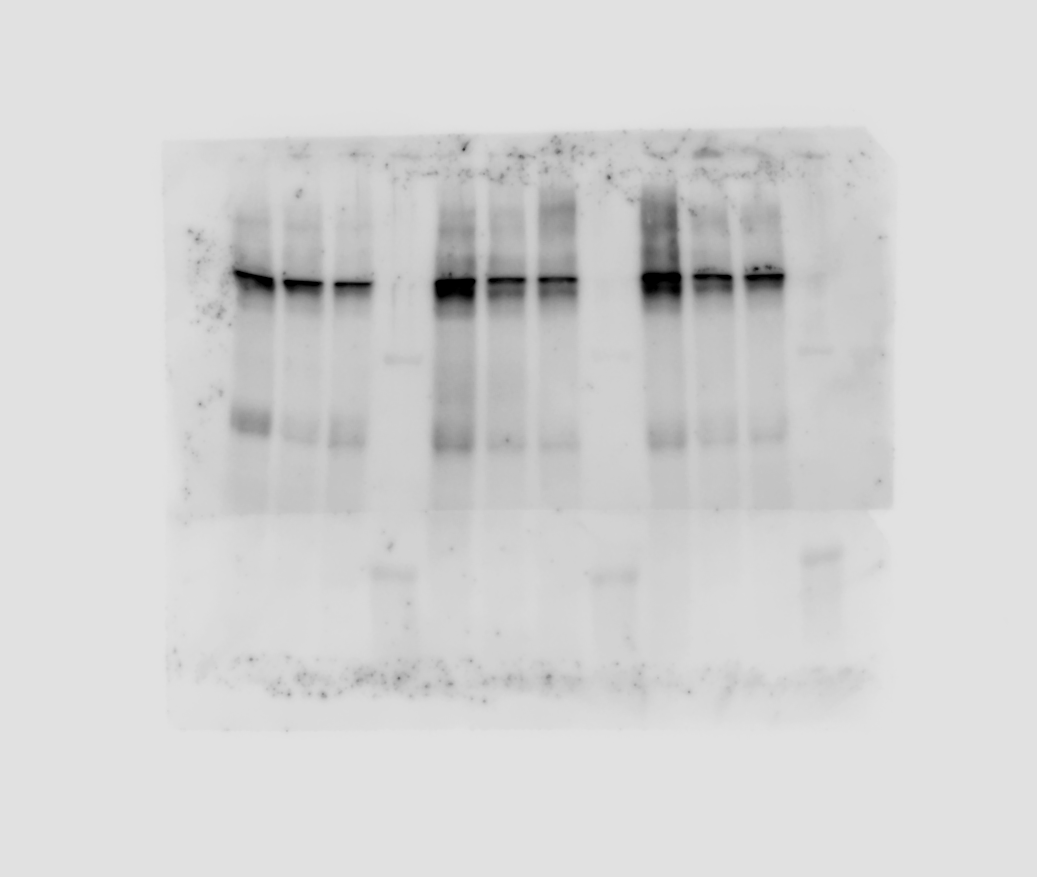

Supplement: Figure 9—source data 3. [file elife-91204-fig9-data3.zip › Figure 9-source data 3-Raw unedited blots/Figure 9-Source Data 3-KCNQ2.tif]

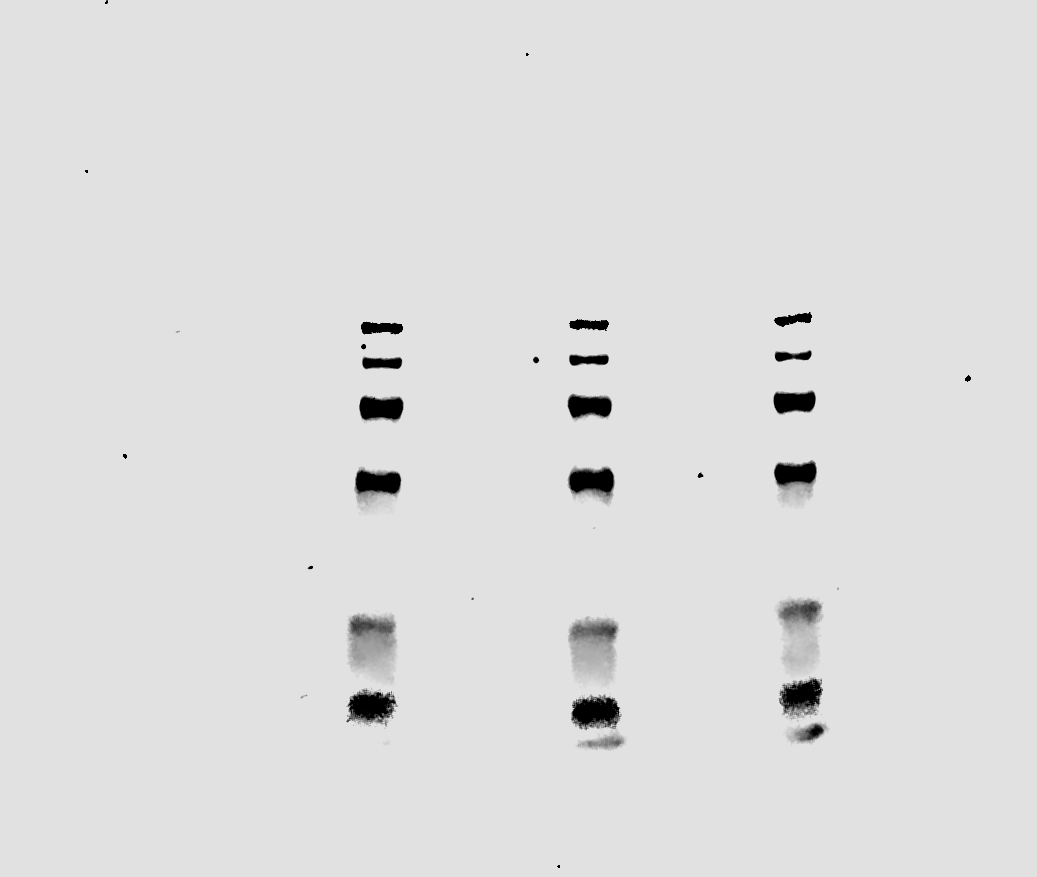

Supplement: Figure 9—source data 3. [file elife-91204-fig9-data3.zip › Figure 9-source data 3-Raw unedited blots/Figure 9-Source Data 3-KCNQ3 MW stds.tif]

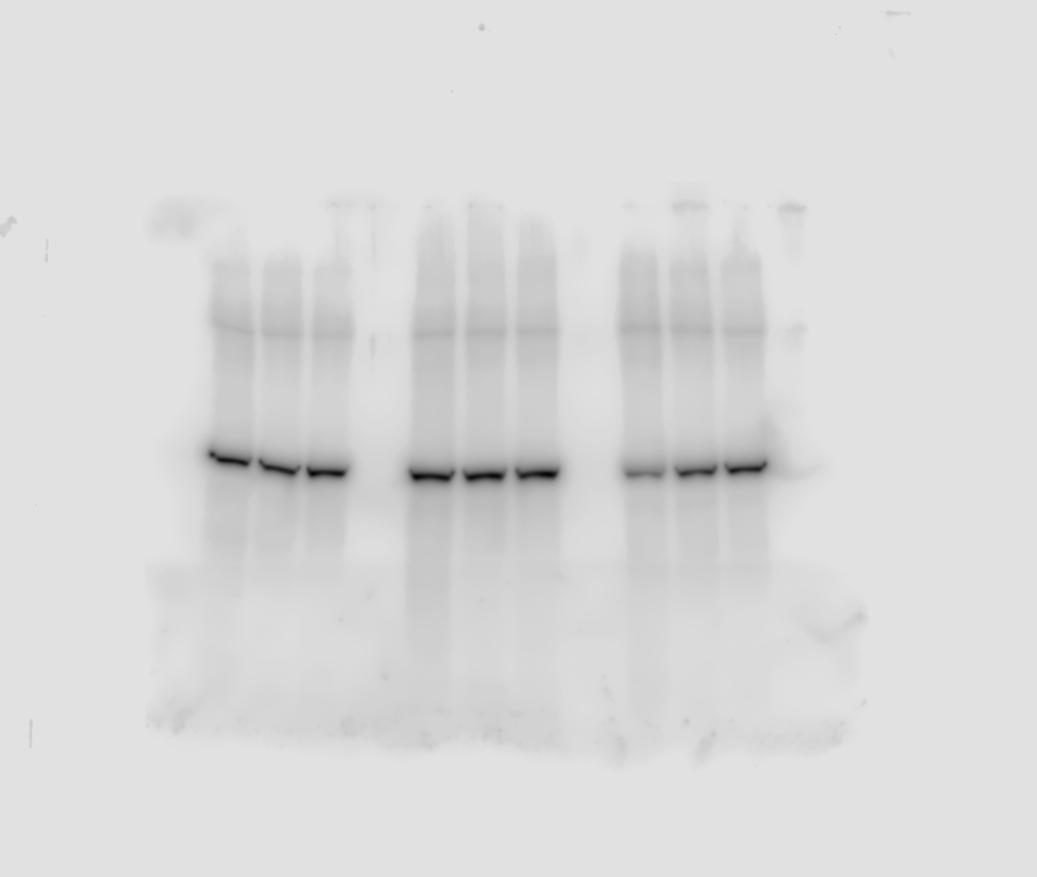

Supplement: Figure 9—source data 3. [file elife-91204-fig9-data3.zip › Figure 9-source data 3-Raw unedited blots/Figure 9-Source Data 3-KCNQ3.tif]

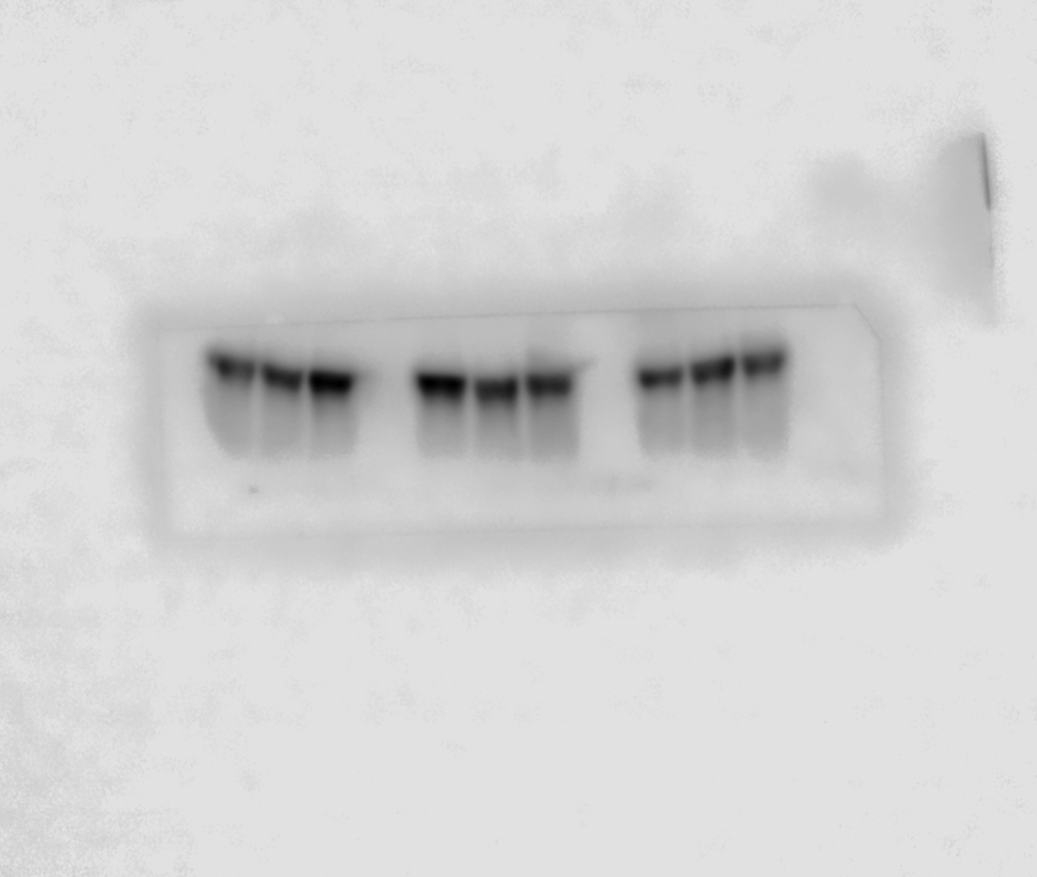

Supplement: Figure 9—source data 3. [file elife-91204-fig9-data3.zip › Figure 9-source data 3-Raw unedited blots/Figure 9-Source Data 3-Tubulin.tif]

WT

G256W/+

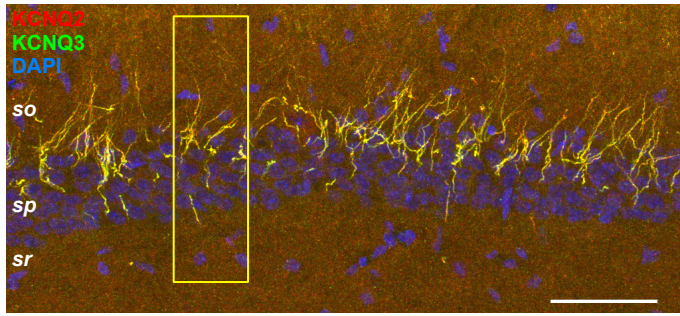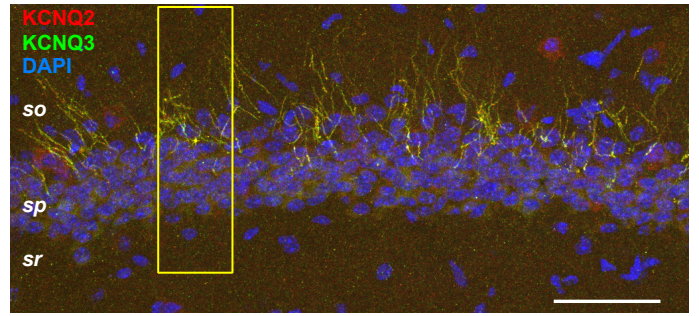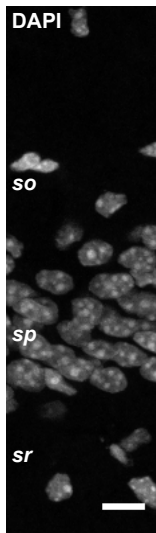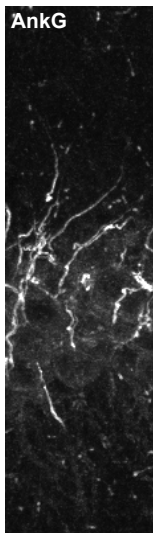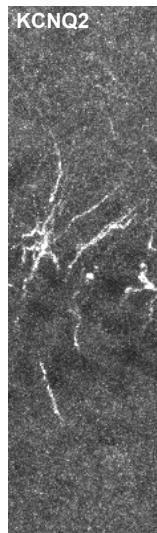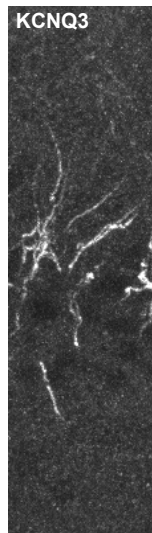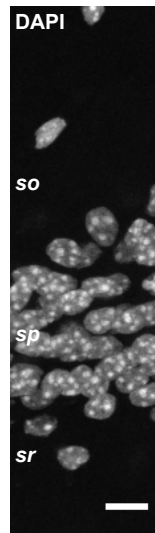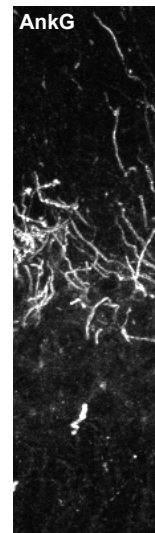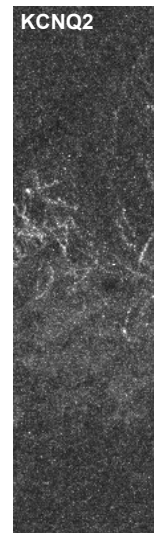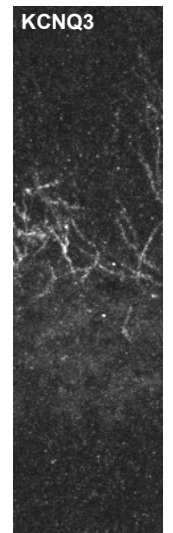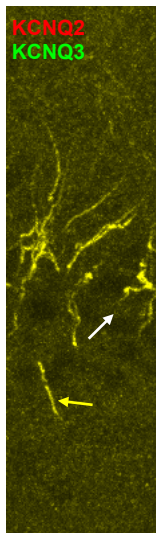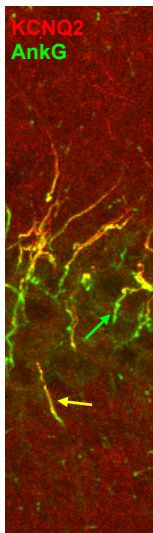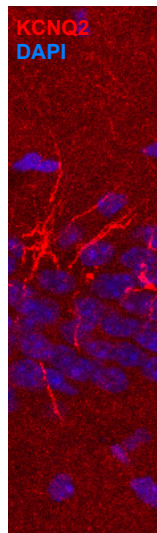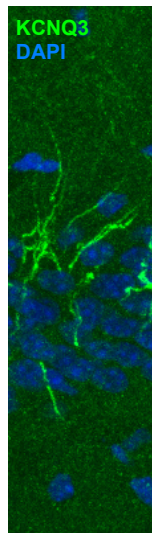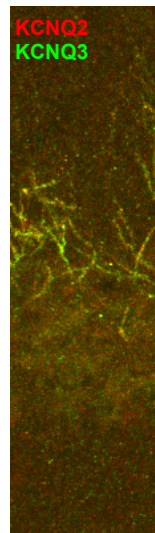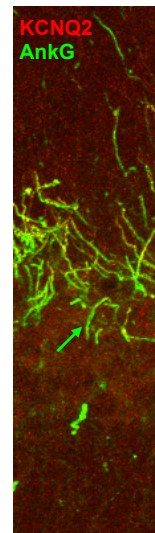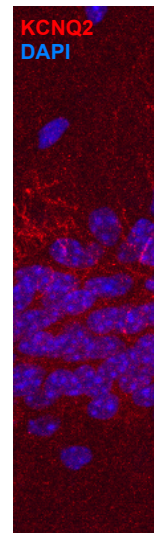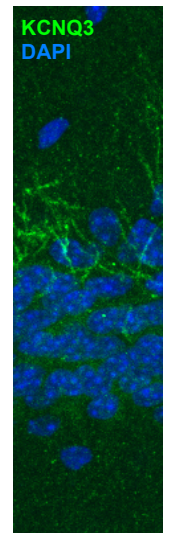

Supplement: Source data 1. — In upper panels, the simultaneously acquired AnkG color channel is not included in the merge, for clarity. In middle and lower panels, the yellow boxed regions of upper panels are shown as single channel greyscale and as the indicated merges of 2 channels. In lower merge images, yellow arrows indicate KCNQ2/KCNQ3 overlap, white and green arrows indicate AnkG-only labeling of proximal AIS. Scales: 50 μm, upper; 10 μm, middle and lower. [file elife-91204-data1.pdf]

WT

E254fs/+

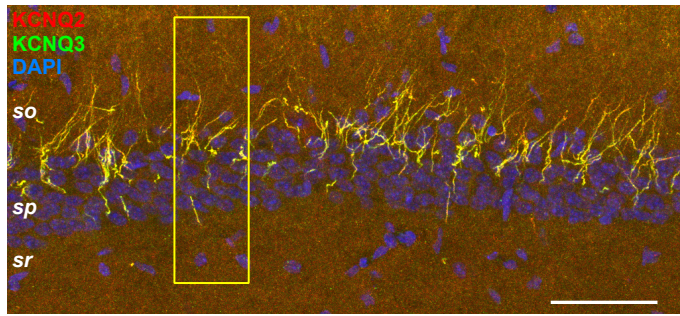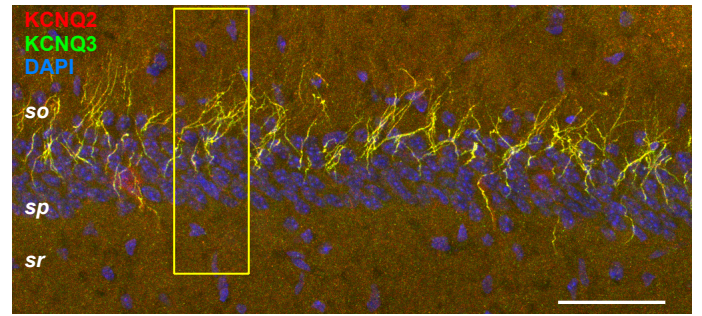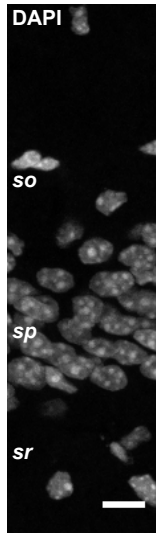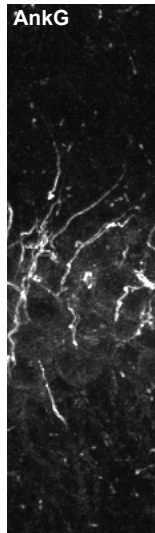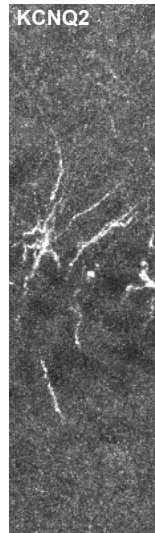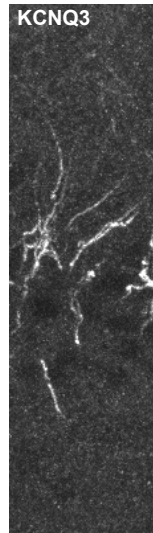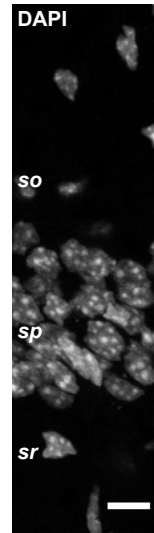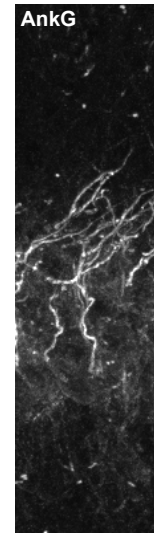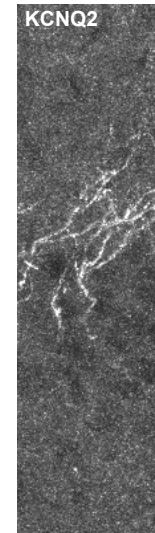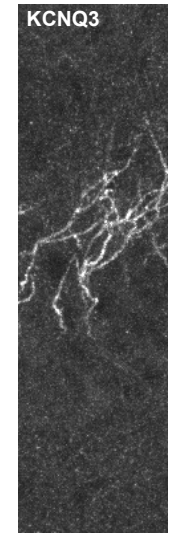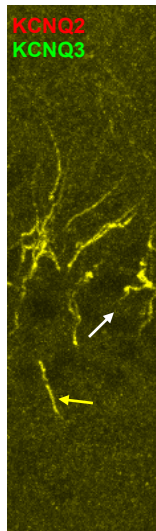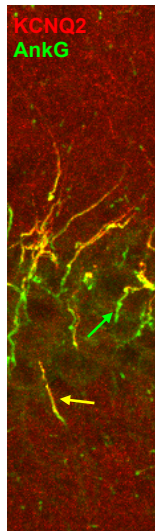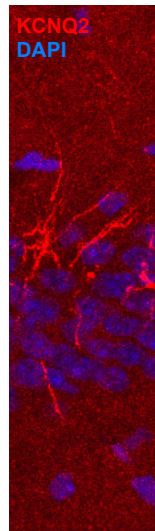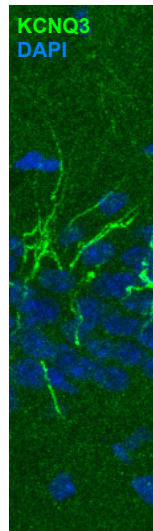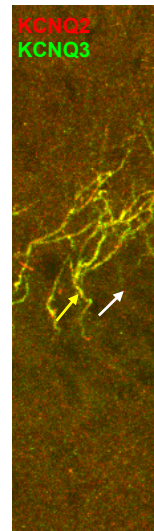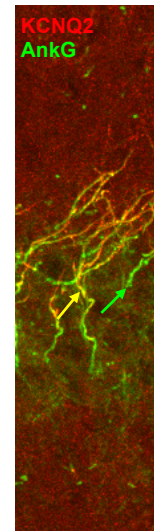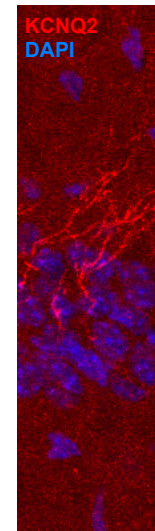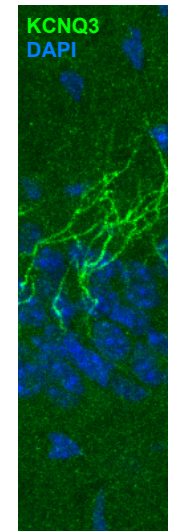

Supplement: Source data 2. — In middle and lower panels, the yellow boxed in upper panels is shown as single channel greyscale and as the indicated merges of 2 channels. In lower merged images, yellow arrows indicate portions of AISs showing KCNQ2/KCNQ3 overlap, and white and green arrows indicate AnkG-only labeling of proximal AISs. Scales: 50 μm, upper; 10 μm, middle and lower. [file elife-91204-data2.pdf]

WT

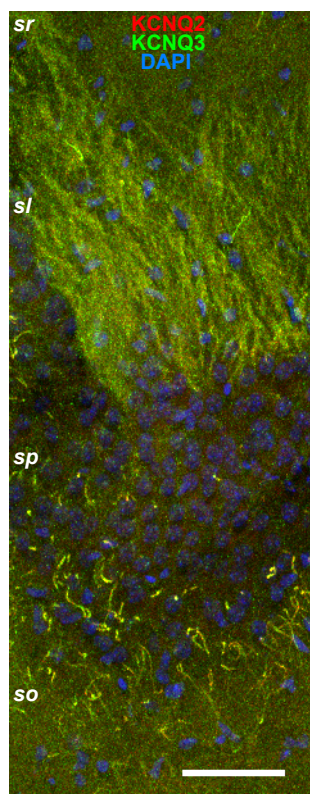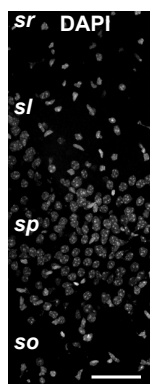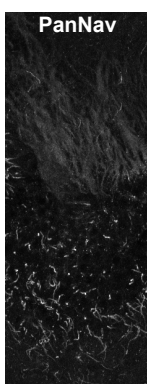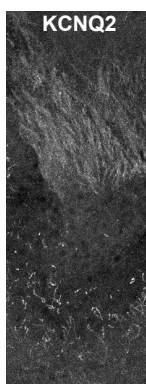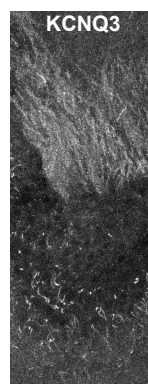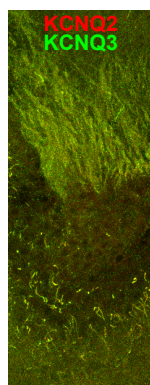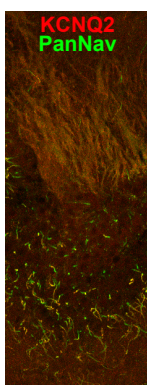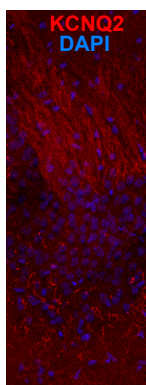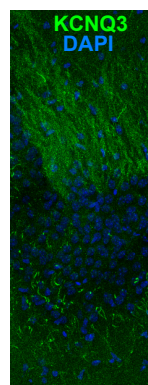

G256W/+

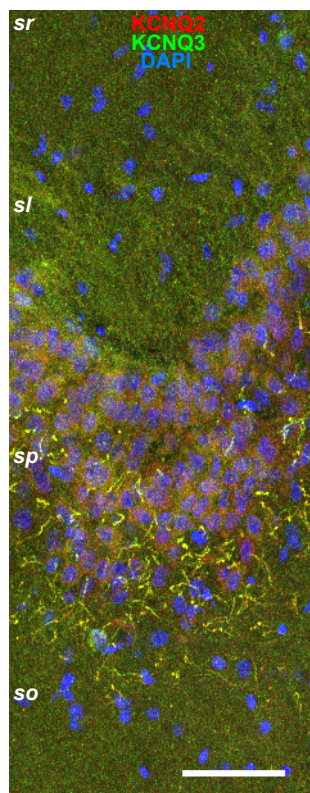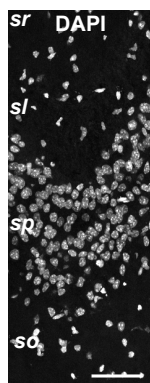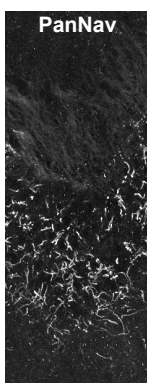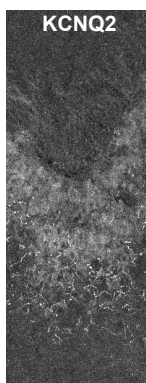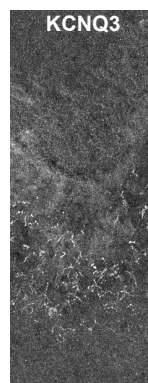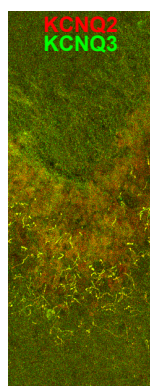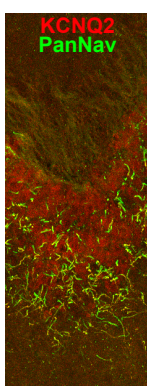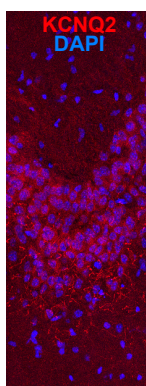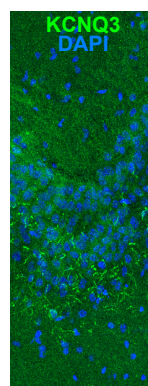

Supplement: Source data 3. — Scales: 50 μm. [file elife-91204-data3.pdf]
